# Molecular cytogenetic characterization of a mosaic small supernumerary marker chromosome derived from chromosome Y in an azoospermic male: A case report

**DOI:** 10.1097/MD.0000000000016661

**Published:** 2019-07-26

**Authors:** Hongguo Zhang, Xiangyin Liu, Dongfeng Geng, Fagui Yue, Yuting Jiang, Ruizhi Liu, Ruixue Wang

**Affiliations:** a Center for Reproductive Medicine and Center for Prenatal Diagnosis, First Hospital; b Jilin Engineering Research Center for Reproductive Medicine and Genetics, Jilin University, Changchun, China.

**Keywords:** azoospermic male, de novo AZFb+c microdeletion, sSMC(Y) mosaicism, Yq deletion

## Abstract

**Rationale:**

Small supernumerary marker chromosomes (sSMCs) can be usually discovered in the patients with mental retardation, infertile couples, and prenatal fetus. We aim to characterize the sSMC and explore the correlation between with sSMC and male infertility.

**Patient concerns:**

A 26-year-old Chinese male was referred for infertility consultation in our center after 1 year of regular unprotected coitus and no pregnancy.

**Diagnosis:**

Cytogenetic G-banding analysis initially described a mosaic karyotype 47,X,Yqh-,+mar[28]/46,X,Yqh-[22] for the proband, while his father showed a normal karyotype. The chromosome microarray (CMA) analysis showed there existed a duplication of Yp11.32q11.221, a deletion of Yq11.222q12, a duplication of 20p11.1 for the patient. Azoospermia factor (AZF) microdeletion analysis for the patient showed that he presented a de novo AZFb+c deletion. Fluorescence in situ hybridization further confirmed the sSMC was an sSMC(Y) with SRY signal, Y centromere, and Yq deletion.

**Interventions:**

The patient would choose artificial reproductive technology to get his offspring according to the genetic counseling.

**Outcomes:**

The sSMC in our patient was proved to be an sSMC(Y), derived from Yq deletion. The spermatogenesis failure of the proband might be due to the synthetic action of sSMC(Y) mosaicism and AZFb+c microdeletion.

**Lessons:**

It is nearly impossible to detect the chromosomal origin of sSMC through traditional banding techniques. The molecular cytogenetic characterization could be performed for identification of sSMC so that comprehensive genetic counseling would be offered.

## 1 Introduction

Small supernumerary marker chromosomes (sSMCs) are described as structurally abnormal chromosomes that cannot be unambiguously identified by conventional banding cytogenetics, and they are generally equal in size or smaller than a chromosome 20 of the same metaphase spread.^[1,2]^ The incidence rate in general population is about 0.3 to 0.5/1000.^[2]^ However, in the patients with fertility problems, the rate of sSMC was elevated to 0.125%, and the occurring frequency of sSMC carriers seems to be male prone than the female (0.165% vs 0.022%).^[3]^ The frequent sSMC are derived from chromosome 15, i(12p), der(22), inv dup(22), and i(18p).^[4]^ Although the karyotype/phenotype correlation between sSMC and male infertility is still unclear, the related reports show that the existence of sSMC is associated with the spermatogenesis impairment, especially in oligoasthenozoospermia.^[3,5]^

As estimated, about 50% sSMC carriers presented somatic mosaicism.^[6]^ The infertility involved in sSMC could result from the duplications of some genes or mechanical effects perturbing meiosis.^[5]^

Azoospermia, entirely loss of sperm ejaculation, takes up the proportion of 10% to 15% in the infertile men^[7,8]^ and 1% of all male.^[9]^ Herein, we present the molecular cytogenetic characterization of mosaicism for an sSMC derived from chromosome Y in an azoospermic male.

## 2 Case report

A 26-year-old Chinese male was referred for infertility consultation in our center after 1 year of regular unprotected coitus and no pregnancy. His height was 170 cm and weight was 70 kg. He presented cryptorchidism and the left and right testicular volume is about 8 mL separately. The development/growth of penis was normal. Moreover, no other abnormal physical examinations were observed. A series of routine examinations were conducted. Semen analysis and levels of sex hormones are listed in Table 1. The male was finally diagnosed as azoospermia according to the semen routine examination.^[10]^ The proband’ father was 49 years old when he was recalled to undergo the azoospermia factor (AZF) microdeletion examination. Our study protocol was approved by the Ethics Committee of the First Hospital of Jilin University, and the informed written consents were obtained from the patients for publication of this case report and accompanying images.

**Table 1:**
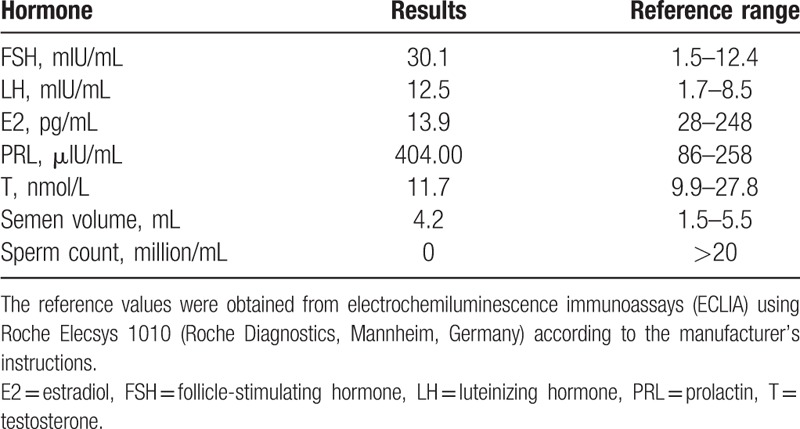
Semen analysis and levels of sex hormones.

## 3 Materials and methods

### 3.1 Karyotype analysis

The G-banding technique was applied on the cultured peripheral blood cells for chromosomal karyotype analysis. Twenty metaphases were analyzed for the patient and his father. We described the karyotype according to the ISCN 2013 nomenclature.^[11]^

### 3.2 Chromosome microarray analysis

Genomic DNA was isolated from 5 mL of peripheral blood of the patient. Then the procedures were conducted through CytoScan 750K array (Affymetrix, Santa Clara, CA). Thresholds for genome-wide screening were set at ≥200 kb for gains, ≥100 kb for losses. The detected copy number variations were comprehensively estimated by comparing them with published literature and the public databases: Database of Chromosomal Imbalance and Phenotype in Humans using Ensemble Resources (DECIPHER), database of genomic variants (DGV), Online Mendelian Inheritance in Man, National Center for Biotechnology Information, and so on.

### 3.3 High throughput sequencing

High-throughput MLPA semiconductor sequencing was applied on the proband and his father to detect the AZF region microdeletions, in which 138 locus-specific oligonucleotides (*loci*) were used as markers. High-throughput MLPA semiconductor sequencing was performed using the reported method.^[12]^

### 3.4 Fluorescence in situ hybridization analysis

Based upon the results above, fluorescence in situ hybridization (FISH) specific for chromosome Y was performed on metaphase slides for the patient to further confirmation through the standard operating protocol (Cytocell Technologies, Cambridge, UK). The detecting probes are as follows: red-labeled sex-determining region Y (SRY) probe with 2 nonoverlapping probes, the green-labeled probe for heterochromatic region (DYZ1) in Yq12 and an alphoid probe for the centromere.

## 4 Results

**Figure 1:**
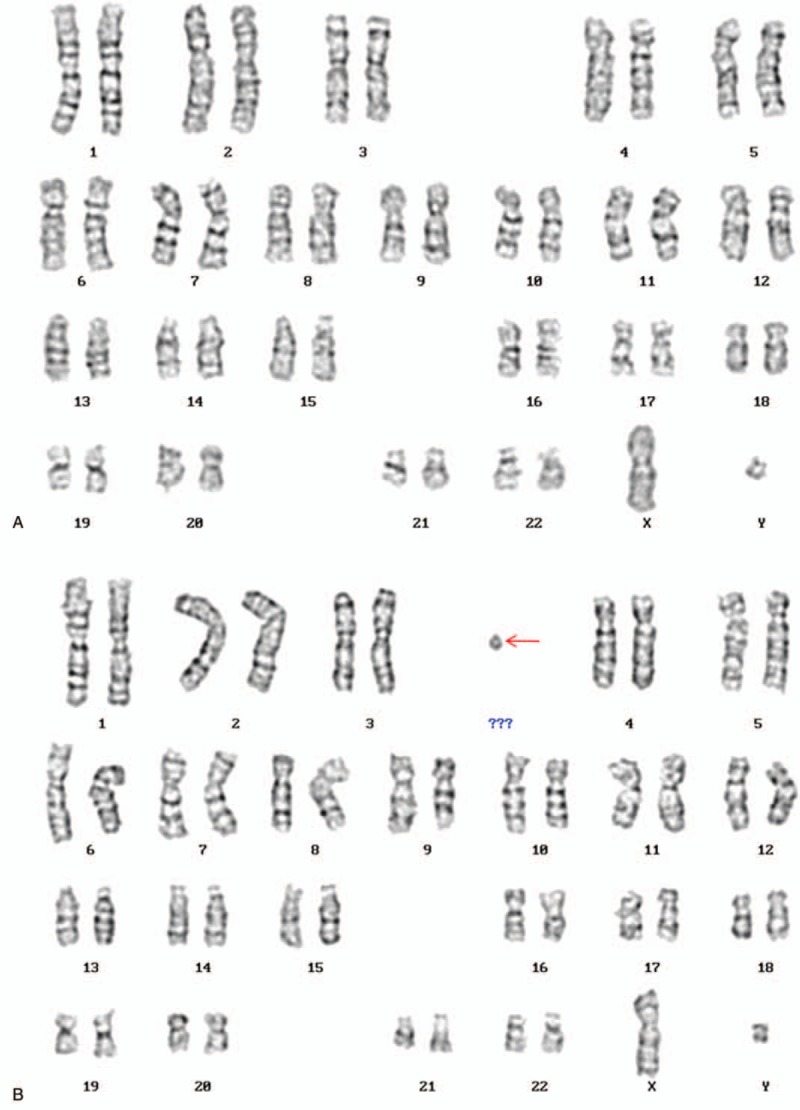
Karyotype of the patient identified by GTG banding technique without small supernumerary marker chromosome (sSMC) (A) and with sSMC (B). Arrow indicated the sSMC.

Cytogenetic G-banding analysis initially described a mosaic karyotype47,X, Yqh-,+mar[28]/46,X,Yqh-[22] for the proband (Fig. 1). To characterize the sSMC for details, the chromosome microarray (CMA) analysis was applied and the results were

**Figure 2:**
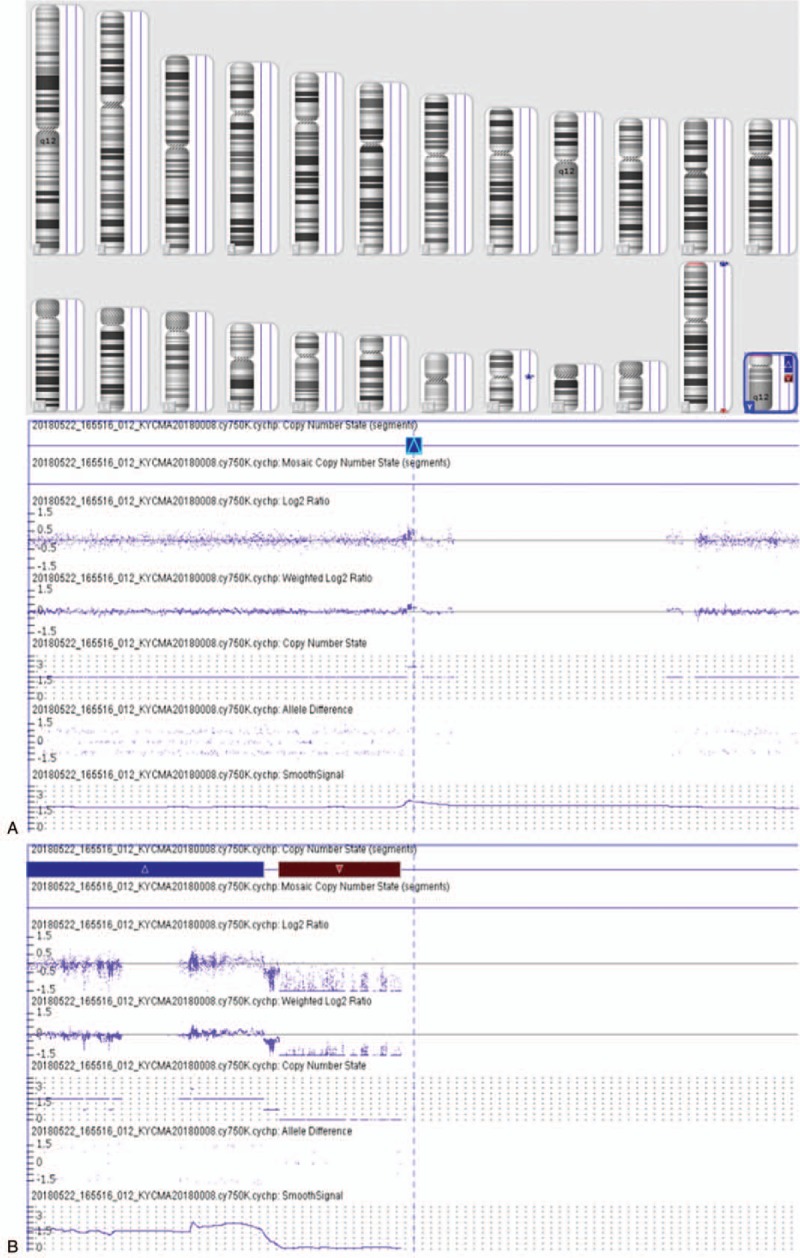
Chromosome microarray on peripheral blood depicted 20p11.1 duplication (A) and Yp11.32q11.221 duplication and Yq11.222q11.23 deletion and Yq12 deletion (B).

**Figure 3:**
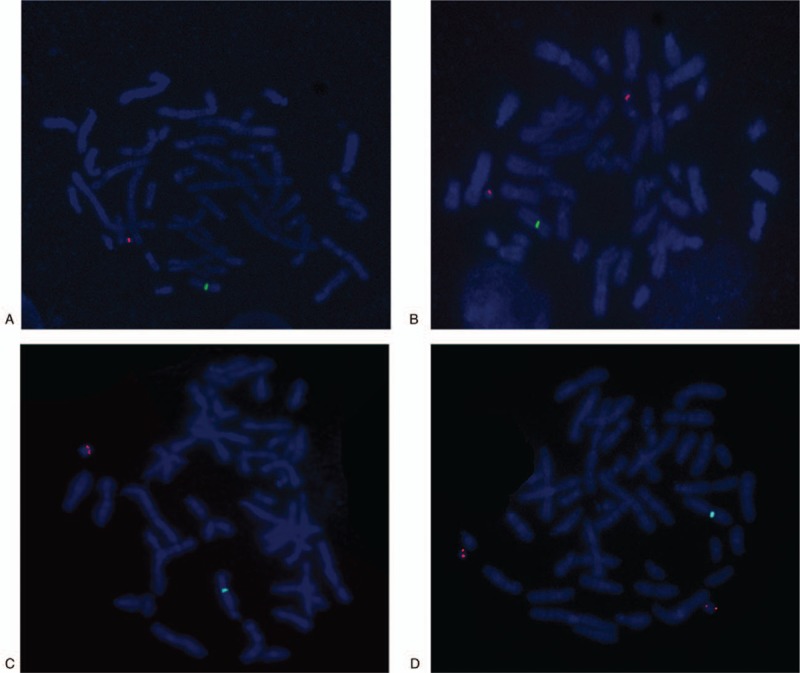
Metaphase-fluorescence in situ hybridization results of an alphoid probe for the Y centromere and SRY probe: (A) 1 centromere signal, (B) 2 centromere signals, (C) 1 SRY signal, and (D) 2 SRY signals.

**Figure 4:**
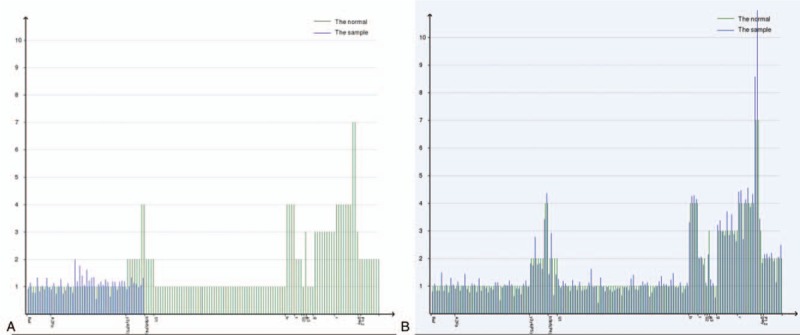
Schematic diagram of Y chromosome microdeletions detected by high throughput sequencing. Horizontal axis represents each locus of azoospermia factor (AZF) region and reference regions, and vertical axis represents normalized copy number of each locus of the AZF region: (A) the patient with AZFb+c deletion, (B) the father.

- arr[hg19]20p11.1(25,624,632–25,839,391) × 3
- arr[hg19] Yp11.32q11.221(118,551–19,556,683) × 2
- arr[hg19] Yq11.222q11.23(20,618,887–28,799,654) × 0
- arr[hg19]Yq12(59,044,874–59,336,104) × 0

which illustrated that there existed a duplication of Yp11.32q11.221, a deletion of Yq11.222q11.23, a duplication of 20p11.1 (Fig. 2). Subsequently, FISH using SRY probe and an alphoid probe for the Y centromere was applied for further verification. The alphoid-specific Y-centromeric probe separately detected 1 signal (Fig. 3A) and 2 clearly distinct signals (Fig. 3B) in 2 cell lines, which caters for the karyotypic mosaic descriptions. The SRY probe detected that there existed 1 SRY signal (Fig. 3C) and 2 SRY signals (Fig. 3D), which inferred the existence of SRY signal in all cell lines. Meanwhile, we failed to detect the green-labeled probe for heterochromatic region, which illustrated that the Yq12 was absent in all cell lines. To further evaluate the infertility and confirm whether the Yq deletion is inherited, we recalled his father back for chromosome karyotype analysis. Meanwhile, they both accepted the high throughput sequencing detecting for AZF microdeletion. The analysis result showed that the father presented a normal karyotype and no microdeletions in AZF region (Fig. 4B), while the proband carried a de novo AZFb+c deletion (Fig. 4A). The patient would choose artificial insemination with donor sperm to get his offspring according to the genetic counseling.

## 5 Discussion

In this study, we described an azoospermic male with a high-level mosaicism of sSMC(Y) derived from chromosomal Y and AZFb+c microdeletion.

The sSMCs, as a specific genetic imbalance, are usually discovered in the patients with mental retardation, infertile couples, and prenatal fetus.^[3]^ The sSMC can display different shapes, for example, the ring, centric minute, and inverted duplication. Among them, the sSMC resulting from acrocentric chromosome would be more easily leading to the infertility.^[5]^ Currently, the genotype–phenotype correlation in sSMC is complicated and unclear. According to the clinic research, 3 factors are mainly responsible for the phenotypes involving sSMC cases: the chromosomal size/origin of the euchromatin, the mosaic proportion of the sSMC, and existence of a uniparental disomy of sSMC's sister chromosomes.^[13]^ According to the review, sSMC(15) was the most frequently reported sSMC associated with oligo- or azoospermia.^[14]^ For sSMC(Y), an unusual anomaly of Y chromosome was inclined to exist in a mosaic form with a 45,X cell line.^[15]^ Manvelyan et al^[16]^ described a female with fertility problems showing 47,XX,+mar/46,XX, with the sSMC(Y) identified as (Y)(::p11.1→?q11.2::). Armanet et al^[5]^ delineated 2 cases with sSMC(Y) in their research review. One was a 41-year-old azoospermia male with 47,XY,+inv dup(Y)(q11.?1). The other was a 27-year-old female for intracytoplasmic sperm injection (ICSI) with 47,XX,+mar, with sSMC(Y) characterized as r(Y)(::p11.1→?q11.2::). In our report, FISH analysis inferred the sSMC of the patient contained SRY signal and Y centromere. Combined with CMA results, the sSMC was identified as sSMC(Y) derived from del Yq, which might be involved in severe infertility. For the infertile sSMC cases with spermatozoa, ICSI could be considered although there exists risk of transmitting the SMC and producing unbalanced offspring after ICSI with a spermatozoon from a man carrying an SMC.^[17]^ Meanwhile, preimplantation genetic diagnosis could detect the sSMC in preimplantation embryos from sSMC carriers as well as aneuploidy in the embryos according to the specific probes.^[18]^ However, prenatal diagnosis should be proposed regardless of assisted reproduction procedures.

As is known, the AZFs regions (AZFa, AZFb, and AZFc), located in the chromosome Yq11, play critical roles in regulating normal spermatogenesis.^[19,20]^ The proband presented a de novo AZFb+c microdeletion, the incidence rate of which is ranging from 8.3% to 14.2% in the infertility male with AZF microdeletion.^[21,22]^ The deletions of AZFb or AZFb+c regions were critical genetic causes of Sertoli cell only syndrome and/or maturation arrest contributing to azoospermia.^[23]^ In our patient, the AZFb+c microdeletion existed in all cell lines, which interpreted the severity of his azoospermia. Due to the limitations of banding resolution and chromosomal Y polymorphism, the chromosomal Y aberration in our report was initially defined as Yqh-, but subsequent molecular testify delineated the Y anomalies were actually delYq, which reminded us to pay more attention to those seemingly Yqh- patients with spermatogenesis failure. And for patients like these, AZF microdeletion screening should be advised for individuals with Y chromosomal abnormalities.

Meanwhile, Yq deletions were significantly involved in sex chromosomal mosaicism and might affect the instability of chromosome Y.^[17]^ Besides, our patient could be approximately regarded as mosaic 47,XYY/46,XY accompanied by Yq deletion. Till now, research of mosaic 47,XYY/46,XY males with fertility problems are inadequate in clinic. Male with mosaic 47,XYY/46,XY could present various degrees of spermatogenesis. Wang et al^[24]^ reported a 31-year-old male with normal semen parameters with karyotype 47,XYY[20%]/46,XY[80%], and they assumed the mosaic 47,XYY/46,XY males did not have a higher rate of monosomic spermatozoa. Lim et al^[25]^ described 2 cases with mosaic 47,XYY/46,XY: one with near normal semen parameters and the other with primary infertility and severe oligoasthenozoospermia. They speculated that the 47,XYY mosaic karyotype may be at risk of producing offspring with a hyperdiploid sex constitution. For our proband, he presented a mosaicism of sSMC(Y) derived from Yq deletion, so his spermatogenesis failure might be caused due to their synthetic actions. The somatic mosaicism forming progress in our proband might be described as follows. Intrachromosomal recombination events between homologous large repetitive sequence block in Yq causes the AZFb+c microdeletions, then subsequent parental nondisjunction during cell division after postzygotic mitosis leads to the extra Y with AZF microdeletion in early embryonic development, which lead to the final mosaicism with Yq deletion.

In addition, there was a 0.22 Mb duplication of 20p11.1 (chr20: 25,624,632–25,839,391) in the CMA results. Considering no OMIM genes existed in this area, the duplicated fragment might have no association with male infertility and be a likely benign variant.

## 6 Conclusion

In conclusion, we identified an azoospermic male with mosaic sSMC(Y) and AZFb+c microdeletion according to the G-banding, CMA, high throughput sequencing and FISH analysis. The cause of the proband's spermatogenesis failure might be due to the combined action of sSMC(Y) mosaicism and AZFb+c microdeletion. Besides, it is nearly impossible to detect the chromosomal origins through traditional banding techniques, so further molecular cytogenetic characterization is necessary for offering better genetic counseling.

## Author contributions

**Conceptualization:** Ruixue Wang.

**Data curation:** Yuting Jiang.

**Formal analysis:** Yuting Jiang.

**Funding acquisition:** Ruizhi Liu.

**Investigation:** Dongfeng Geng.

**Methodology:** Xiangyin Liu.

**Project administration:** Ruizhi Liu.

**Software:** Fagui Yue.

**Validation:** Ruizhi Liu.

**Visualization:** Ruixue Wang.

**Writing – original draft:** Hongguo Zhang.

**Writing – review & editing:** Ruixue Wang.
